# Experimental Study on Abrasive Flow Polishing of Grooves and Oil Holes of Aircraft Engine Main Bearing

**DOI:** 10.3390/mi16101139

**Published:** 2025-10-01

**Authors:** Qinghao Zhang, Jikun Yu, Mingyu Wu

**Affiliations:** School of Mechanical and Power Engineering (ECUST), Dalian Ocean University, Dalian 116023, China

**Keywords:** abrasive flow, durability, controlled manufacturing, aircraft bearings, surface roughness

## Abstract

This study addresses the challenges in machining the raceways and oil holes of aircraft engine bearing rings by conducting abrasive flow machining experiments on main bearing rings which had undergone ultra-precision grinding. Viscoelastic abrasive media containing cubic boron nitride of different particle sizes is used during the experiments. The results show that bearing performance is improved significantly in terms of surface roughness and residual compressive stress consequently; the overall surface quality is raised. The machining process meets the precision requirements for the main bearings of this type of aircraft engine, validating the feasibility and effectiveness of Abrasive Flow Machining (AFM), and the foundation for further optimization of this process is set through this research.

## 1. Introduction

With the increasing demands for high performance and extended service life in modern aero-engines, the manufacturing precision and surface quality of the main bearing rings have become critically important factors. Aero-engines operate under highly complex conditions, in which bearings are required to function reliably under high temperatures, high speeds, and heavy loads for prolonged periods [1,2]. As a critical rotating component in aero-engines, the bearing ring must not only withstand significant mechanical stresses but also exhibit exceptional fatigue resistance as well as wear durability [3]. Particularly, the groove of the bearing ring, which directly contacts the rolling elements, requires exceptionally high surface finish to minimize friction and wear. Consequently, the overall performance of the bearing is significantly influenced by the surface roughness and residual stress of the groove [4]. Additionally, the oil holes on the bearing ring play an important role in the lubrication system, where any residual machining burrs or contaminants could directly damage the lubrication efficiency and overall reliability of the bearing [5]. Therefore, enhancing the surface machining quality of bearing ring grooves and oil holes is considered to be a significant challenge in the manufacturing of aircraft engine bearings.

The machining challenges of main bearing rings are primarily reflected in the following aspects: First, the groove surface is required to achieve high precision and low roughness and maintain minimal residual stress distribution to enhance fatigue resistance and service life. Second, the complete removal of burrs and the finishing treatment of oil holes are critical, as they not only affect lubrication efficiency but also directly influence the operational reliability of the bearing. Furthermore, the complex geometries and the inherent difficulty of machining materials further aggravate the process difficulty.

Traditional polishing methods include abrasive belt polishing, mechanical polishing, vibratory polishing, electrolytic polishing, abrasive flow polishing, chemical mechanical polishing, honing, magnetorheological polishing, and so on [6,7,8,9,10,11,12]. Various polishing methods exhibit distinct advantages and limitations in practical applications. Considering factors such as process consistency, precision, limitations, and cost, the industry has introduced an advanced finishing technology: abrasive flow machining (AFM) [13,14,15]. As an emerging precision machining technology, abrasive flow polishing demonstrates significant advantages in enhancing surface quality and reducing residual stresses [16,17].

This study aims to explore the feasibility and superiority of abrasive flow polishing technology in the practical application of machining the channels and oil holes of aerospace engine main bearing rings. The paper first introduces the basic principles of abrasive flow polishing and designs an experimental plan for the abrasive flow processing of bearing ring channels and oil holes. Subsequently, key parameters such as surface roughness, residual stress, and minimum oil film thickness are measured and analyzed, ultimately providing theoretical and experimental insights into the improvement of bearing performance.

## 2. Experiment Preparation

**Figure 1:**
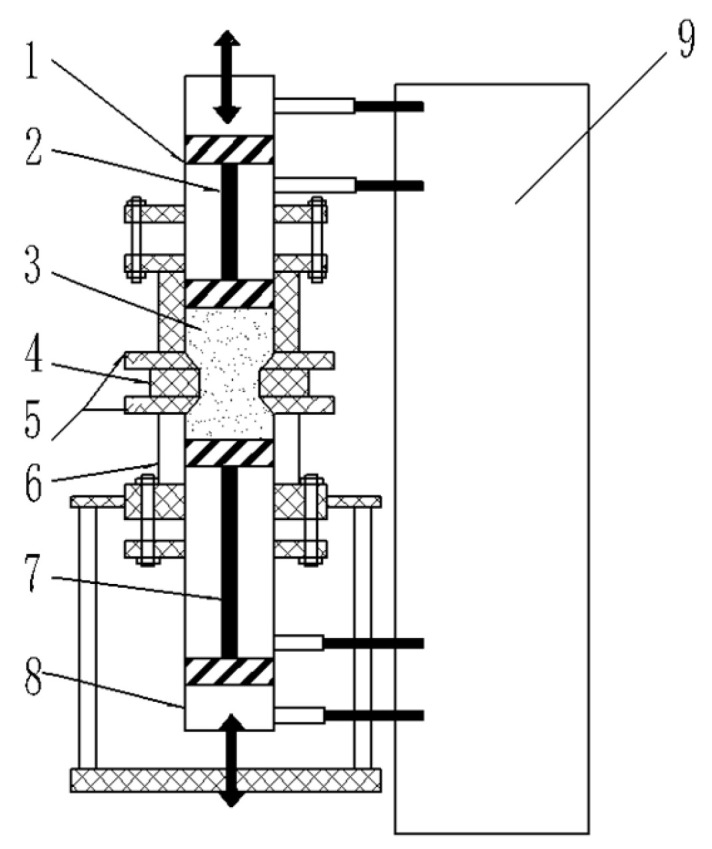
Equipment and schematic diagram. 1—Piston Cylinder; 2—Piston; 3—Abrasive Medium; 4—Workpiece to Be Processed; 5—Fixture; 6—Abrasive Cylinder; 7—Piston; 8—Piston Cylinder; 9—Hydraulic System.

Abrasive flow machining was first proposed in the United States in the 1960s, and the related literature and patents were formed in the 1980s [18,19]. This technology is a flexible precision machining technique that utilizes a viscoelastic medium that contains abrasives to perform micro-cutting and grinding on the surface of workpieces through channels formed by specialized fixtures under pressure. The machining principle is illustrated in Figure 1 [20]. In abrasive flow machining, the viscoelastic medium containing abrasives flows uniformly through the workpiece clamped by the fixture under pressure. At the same time, multiple and repeated extrusion and friction can be performed [21]. The complex geometric shapes of the main bearing raceway and oil holes make it difficult for traditional mechanical grinding to reach micro-surface unsmoothness. However, the abrasives in abrasive flow machining can access these hard-to-reach areas, address burrs and reduce the roughness of the raceway and oil holes. Compared to traditional ultra-precision machining, the flexible medium in abrasive flow machining ensures that abrasives act uniformly on the surface of the workpiece, whether it is the curved surfaces of the raceway or the inner walls of the oil holes. This kind of non-contact machining method avoids scratches caused by contact with the workpiece. Additionally, the abrasives are evenly distributed during the flow process so that consistency and uniformity of the machined surface are ensured, thereby reducing partial over-processing or under-processing [22,23].

Traditional machining methods may leave tiny cracks or defects on the workpiece surface, which can serve as initiation points for fatigue cracks during bearings operation time, thereby reducing the service life of the bearings. Abrasive flow machining effectively eliminates these micro-defects through its flexible machining characteristics; as a result, a more uniform and smoother workpiece surface is produced which reduces stress concentration points and enhances the “controllability” of the residual compressive stress in the workpiece [16]. All these significantly improve the fatigue resistance of the main bearings.

### 2.1. Experimental Equipment and Materials

**Figure 2:**
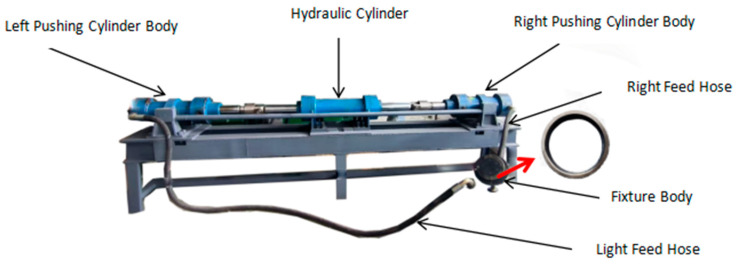
Horizontal AFM Experimental Platform.

The experimental subject is a main bearing ring made of 8Cr4Mo4V material, with a hardness of HRC61. The dimensions of the bearing ring are D × d1 × d2 × B = 200 mm × 186 mm × 178 mm × 35 mm. The surface roughness Ra of the raceway is 0.32 μm, while the surface roughness Ra of the oil holes is 2.198 μm. A self-developed MLL250 horizontal abrasive flow polishing machine is used as the abrasive flow polishing experimental platform, as shown in Figure 2. Customized fixtures designed for the main bearing ring and self-formulated viscoelastic cubic boron nitride abrasives with particle sizes of 10,000 and 18,000 mesh are used for processing the raceway. Considering the small diameter of the oil holes, coarser abrasives could lead to hole enlargement; therefore, 5000-mesh viscoelastic cubic boron nitride abrasives are chosen for oil hole processing.

The ultra-precision machining requirements for this type of aircraft engine main bearing are as follows:

1. Surface roughness for the raceway is required to be within Ra 0.025 µm, with significant improvement in microtopography;
2. Surface roughness for the oil holes is required to be within Ra 0.8 µm;
3. Compressive stress on the raceway surface is required to be greater than −450 MPa.

### 2.2. Measurement of Experimental Data

Surface roughness is a key indicator of the surface quality of a workpiece, while residual compressive stress is an important parameter for assessing the internal stress distribution within the material during the machining process, which will directly affect the material’s fatigue performance and service life. Prior to abrasive flow machining, three measurement points were selected at 120° intervals along the circumferential direction at both ends of the bearing ring to measure its hardness, raceway roundness, profile, arithmetic roughness (Ra), root mean square roughness (Rq), maximum valley depth (Rv), maximum height difference (Rt), and average height of the ten points (Rz). The surface compressive stress was tested with the nanoindentation method. The surface roughness was measured using a Talysurf 120 mm PGI surface profilometer (Taylor Hobson, Leicester, UK). The residual stress was measured with D8 discover X-ray Diffraction (XRD, Bruker AXS, Karlsruhe, Germany). The specific measurement results are shown in Table 1.

### 2.3. Design of the Fixture

**Figure 3:**
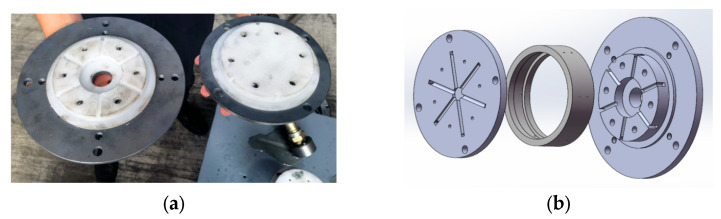
Fixture photograph and its 3D model. (**a**) Photograph of the fixture. (**b**) 3D model of the fixture.

During the abrasive flow machining process, it is essential to design a suitable fixture to ensure that the abrasives are guided by the medium flow channel restrictor to compress the processed area, while other parts of the bearing ring raceway will not be influenced. In this study, a single fixture was chosen to accomplish the machining tasks for both the oil holes and the raceway of the aircraft engine main bearing ring. Given that the roughness requirement for the oil holes is lower than that for the raceway, the designed fixture maintains a gap about 2–3 mm between itself and the main bearing ring to ensure the effectiveness and precision of the machining process [24]. The design of the fixture is shown in Figure 3.

Under high pressure, the flowing abrasives are injected into the fixture system from the outlet. After being guided through the channel design of the fixture and acting on the machining area, the abrasives are expelled from another outlet under the guidance of the the opposite side of the fixture channel. During the machining of the oil holes, the flow is unidirectional, whereas multiple processings are conducted in a circulation mode during the machining of the raceway.

### 2.4. Configuration of Abrasives

In the process of abrasive selection, different types of abrasives, particle sizes, densities, and viscosities of the carrier must be chosen based on the material of the workpiece to achieve the desired grinding effect. The material of the aircraft engine’s main bearing is 8Cr4Mo4V, with a hardness of HRC61. In China, this is a widely used high-temperature bearing steel in aircraft engine main shaft bearings, characterized by high strength, high hardness, heat resistance, corrosion resistance, and hardenability.

Cubic boron nitride (CBN) possesses extremely high hardness, second only to diamond, and exhibits excellent grinding performance for high-hardness materials. It has good chemical stability and can maintain its physical and chemical properties at high temperatures, making it resistant to degradation or structural changes. Furthermore, CBN can effectively reduce the wear of the abrasives and the frequency of replacement. Considering factors such as grinding quality, efficiency, and cost, common viscoelastic cubic boron nitride abrasives were selected as the grinding medium for the experiments.

In abrasive flow machining, the base carrier plays a crucial role since it directly affects the effective delivery of abrasives and the machining results. Therefore, selecting a base carrier like styrene-butadiene rubber, which has excellent stability, wear resistance, and flowability is essential. Styrene-butadiene rubber not only resists wear but also exhibits good chemical resistance, allowing it to withstand the oil components in the fluid carrier during the machining process, thus preventing dissolution or degradation.

The binder is used to combine the abrasives and other components, forming a uniform abrasive distribution that ensures the stability and uniformity of the abrasives during the machining process.

Lubricants can effectively reduce the friction generated during the grinding process, minimizing wear between the abrasives and the workpiece surface.

Dispersants can improve the dispersion of abrasives in the liquid, ensuring that the abrasives are uniformly distributed in the processing medium, thereby preventing sedimentation or agglomeration of the abrasives.

Stabilizers can enhance the fluidity of the processing liquid, ensuring that the abrasives can flow and be transported smoothly during the machining process. The characteristics and composition of the configured abrasives are listed in Table 2.

## 3. Experimental Results and Analysis

With the main bearing ring that has already undergone ultra-precision finishing by the oil stone vibration method, grinding experiments were conducted using the self-developed MLL250 horizontal abrasive flow polishing machine. Two hydraulic cylinders are employed by the abrasive flow machine to symmetrically and reciprocally compress a semi-solid fluid abrasive medium. By passing the viscoelastic medium containing abrasives under high pressure through the channels or holes of the workpiece, the abrasives perform micro-cutting and polishing on the surface, effectively removing micro-unsmoothness and enhancing surface smoothness. Complex shapes can be handled through this process while additional thermal or mechanical stresses can also be avoided.

Initially, a 5000-mesh abrasive flow was used to treat the oil holes of the main bearing ring. Once the roughness of the oil holes met the standard, with an Ra value below 0.8 µm, suitable plugs were used to seal all the oil holes. Subsequently, finer 10,000-mesh and 18,000-mesh abrasive flows were employed for processing the raceway. This design aims to achieve efficient machining of the raceway and holes in the main bearing ring while meeting the stringent requirements for surface roughness.

### 3.1. Experimental Parameter Settings

When machining the main bearing ring, the bearing is first securely fixed in the fixture to ensure the stability of the machining process. The semi-solid abrasive flow medium is delivered to the fixture cavity through the abrasive cylinder. Under the uniform compression of the hydraulic cylinders, the hydraulic system applies uniformly to let the abrasive flow to form a closed flow path around the oil holes. Within this flow path, the abrasives undergo high-frequency friction and polishing against the walls of the oil holes. After, machining plugs are used to seal the oil holes, allowing the abrasive flow to be extruded along the closed flow path formed by the fixture and the outer raceway of the main bearing. This facilitates repeated friction on the surface of the bearing raceway, achieving the desired effect of extrusion and polishing on the raceway channel. The machining parameters are listed in Table 3.

### 3.2. Experimental Results

After machining, the work-piece was cleaned to remove any residual abrasives and liquids. Subsequently, surface roughness and residual stress measurements were conducted to assess the machining effectiveness.

In this study, the structural design of the main bearing ring for the aircraft engine includes 12 oil holes. Based on the controllability, uniform distribution, and representativeness of the data, six of these oil holes were selected for the abrasive flow machining experiments. The smoothness of the oil holes has improved, with its Ra value decreasing from 2.198 µm to 0.641 µm, meeting the requirement of being below 0.8 µm. The roughness and internal diameter differences of the oil holes before and after machining are shown in Table 4. The internal diameter differences of the oil holes before and after machining met the requirements, demonstrating that it was the surface micro-topography rather than the original geometric tolerances of the work-piece been altered by the fluid polishing.

**Figure 4:**
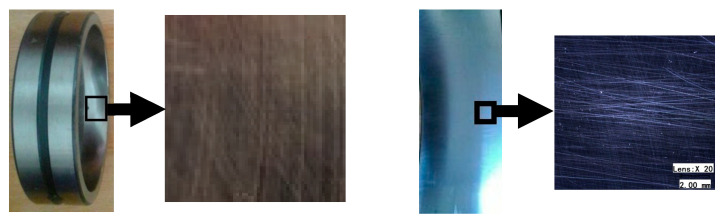
The surface and texture map before and after abrasive flow machining.

After processing with 10,000-mesh cubic boron nitride abrasives, the surface texture of the bearing ring channels was observed with the VHX-600E ultra-depth-of-field 3D microscope. It was found that the channel surface texture had become further chaotic based on the initial ultra-precision ground texture. After processing with 18,000-mesh cubic boron nitride abrasives, the surface roughness of the bearing channels was measured to reach approximately 0.019 µm, representing a significant improvement compared to the surface roughness of 0.032 µm measured before the experiment. The surface and texture of the bearing raceway before and after abrasive flow machining are shown as Figure 4.

In this experiment, the nanoindentation method was employed by using the AntonPaar NHT nanoindentation instrument (Anton Paar GmbH, Graz, Austria) to measure the residual stress of the bearing after abrasive flow machining. Utilizing the Suresh model, the study revealed that the residual compressive stress on the surface of the bearing channels after the experiment was −702 MPa, which is a significant progress compared to the residual compressive stress of −312 MPa measured before abrasive flow machining. The measurement records for the bearing ring channels after the experiment are presented in Table 5.

### 3.3. Results Analysis

#### 3.3.1. Abrasive Flow Machining Mechanism

**Figure 5:**
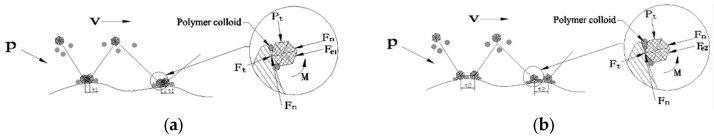
Schematic diagram of the material removal mechanism in abrasive flow polishing. (**a**) Traditional abrasive flow. (**b**) High elastic abrasive flow.

The mechanism of abrasive flow machining operates by applying compressive forces to a fluid medium that contains abrasives and possesses properties as viscoelastic, soft, and cutting properties, forming a semi-solid yet flowable “extrusion block”. This block is then made to flow rapidly and reciprocally over the surface to be machined, performing micro-cutting on the work-piece surface through a process known as “dynamic micro-edge cutting”. Through the complement of the cycle of “extrusion—slip—shear”, metallic material can be removed. A further analysis of the extrusion process reveals that during the flow through the channel, the abrasive particles encapsulated in the high-elasticity colloid serve as the working medium. The extrusion process can be viewed as the work performed by the expansion of the elastic medium, as illustrated in Figure 5. Under the influence of the deformed high-elasticity abrasives, the deformation of the medium experiences a cycle of “gradually increasing—maintaining—then returning to the original state”. Compared to inelastic abrasive media, this process allows for a prolonged time during which the abrasives expand and perform work. In comparison to the ”inelastic” sliding machining time t1, the “elastic” sliding time t2 with viscoelastic abrasives involved in the cutting is greater, specifically t2 > t1. As the time increases, so does the cutting distance, and thus the efficiency of the abrasive “micro-edge” cutting is enhanced. That is to say, under the influence of the high-elasticity colloid, the duration during which the abrasives remain adhered to the machined surface is prolonged, effectively extending the plowing phase, thus stabilizing the “cutting” effect of the same abrasive particles. The specific machining principle can be further elaborated. As shown in the localized magnified view, a single abrasive particle experiences a pushing force Pt from the piston that drives the abrasives forward. The abrasive particles are subjected to two types of normal forces: one is the normal force Fn exerted by the compressed abrasive, and the other is the elastic force Ft from the compressed colloid. Since the elastic force Ft2 of the high-elasticity colloid is greater than the traditional colloid’s elastic force Ft1, the abrasives are subjected to a larger normal force, making it easier for them to penetrate the work piece. Under the pushing force Pt, a plowing effect occurs. As the elastic force increases, the duration of the plowing phase is extended, resulting in smoother and more uniform machining, and the machining efficiency is improved simultaneously [25].

#### 3.3.2. Roughness Analysis

After the initial processing, it is necessary to inspect the roughness and surface condition of the channels. The roughness of the channel surfaces has significantly decreased. Based on the evaluation results after the initial processing, the processing parameters are adjusted. A second inspection of the roughness and surface condition is conducted to assess the effects of the secondary processing. By comparing the results with those of the initial processing, the improvement in surface quality due to the machining can be observed clearly.

After, the initial processing, along with adjustments to the parameters based on the results, help to improve both processing efficiency and effectiveness. By combining the results from both processing stages, a comprehensive understanding of the impact of abrasive flow machining on channel surface roughness can be obtained, allowing for further optimization of the processing technology. The surface roughness can be calculated using Equation (1) [26]:

(1) $$Ra=\frac{1}{A}\iint_{A}\left|z\left(x,y\right)\right|dxdy$$

where the surface height function represents the surface height z(x,y) at a given coordinate and reflects the micro-morphological characteristics of (x,y) the surface; A is the total area of the calculation region, referring to the effective working area of the channel surface; the double integral is used to calculate the sum of the absolute heights of all points within the region, which comprehensively reflects the distribution of surface heights.

In this experiment, the value of A is fixed. After abrasive flow machining, the high points and burrs on the surface are effectively removed, resulting in a significant reduction in the absolute value of z(x,y). According to the formula, the final calculated value Ra will decrease accordingly which indicates that the surfaces roughness after machining is significantly lower than that before machining. After multiple machining processes, the high points and burrs on the surface are effectively removed, and the micro-morphology of the surface tends to stabilize, leading to a smaller variation in ∣z(x,y)∣, which causes the integral result to stabilize. That is, as the number of machining cycle increases, the variation in surface roughness tends to become constant.

#### 3.3.3. Residual Stress Analysis

The nanoindentation method is a widely used technique for testing mechanical properties in micro-regions, particularly suitable for measuring local residual stress in high-hardness materials. Based on the Oliver–Pharr method, the determination of the material’s elastic modulus and hardness can be accessed through the analysis of the force–displacement curve during the indentation process, which can subsequently be used to derive the residual stress. Due to the high hardness (HRC61) of the 8Cr4Mo4V material used in the main bearing, it is essential to strictly control the applied load during testing to ensure that the indenter does not cause irreversible damage to the high-hardness surface. Based on expression (2), an analysis can be performed on the increase of residual compressive stress:

(2) $$\sigma_{r}=H\cdot \left(\frac{1}{\sqrt{2}}\cdot \frac{E_{r}}{H}-1\right)$$

(3) $$\frac{1}{E_{r}}=\frac{1-v_{i}^{2}}{E_{i}}+\frac{1-v^{2}}{E}$$

where $\sigma_{r}$ is residual stress; *H* is material hardness; $E_{r}$ is effective elastic modulus measured by nanoindentation method, $E_{r}$ is the definition formula which is given as (3); $E_{i}$ and $v_{i}$ are the elastic modulus and Poisson’s ratio of the indenter, respectively; *E* and *v* are the elastic modulus and Poisson’s ratio of the material, respectively.

**Figure 6:**
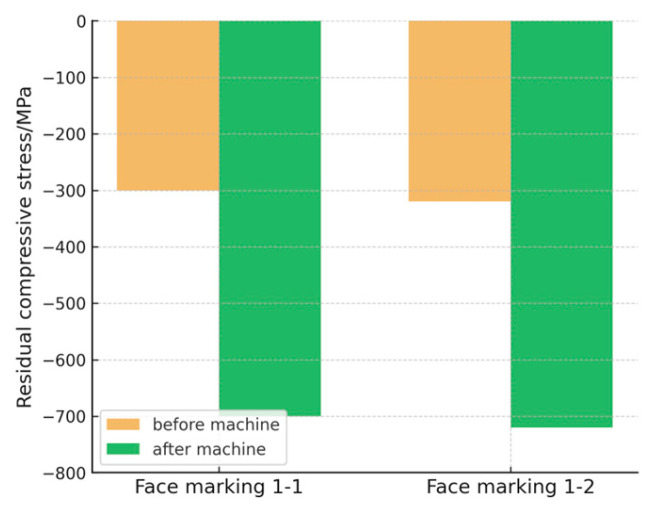
The gradient distribution of residual compressive stress after machining.

In this experiment, abrasive flow machining is adopted. As the process progresses, the material surface will experience the effects of high pressure and shear force. The increase in material hardness H directly contributes to the rise in residual compressive stress $\sigma_{r}$. Meanwhile, under the influence of high pressure and high shear force, the material local structure is changed by its plastic deformation, leading to variations in the effective elastic modulus $E_{r}$, which further enhances the residual compressive stress. Furthermore, existing studies indicate that residual compressive stress can improve bearing life under experimental conditions of normal temperature and corrosive environments [27]. The gradient distribution of residual stress after machining 8Cr4Mo4V is shown in Figure 6.

#### 3.3.4. Analysis of Oil Film Thickness

In elastohydrodynamic lubrication, the formation of oil film thickness is influenced by a combination of effects of load, speed, material properties, and the geometry of the contact surfaces. According to the classical minimum oil film thickness formula proposed by Hamrock and Dowson, the minimum oil film thickness $h_{min}$ is primarily determined by the following factors [28]:

(4) $${h_{min}=3.63U}^{0.68}G^{0.49}W^{-0.073}\left(1-0.68e^{-0.68k}\right)$$

(5) $$k=1.03{\left(\frac{R_{y}}{R_{x}}\right)}^{0.64}$$

(6) $$G=\frac{E^{\prime }}{\left(1-v^{2}\right)}$$

where $h_{min}$ is minimum oil film thickness; U is dimensionless velocity parameter, representing the relative speed of surface motion; G is dimensionless material parameter, indicating the elastic properties of the material; *W* is dimensionless load parameter, reflecting the influence of load on oil film thickness; *k* is geometric parameter, representing the curvature ratio of the contact surface shape; $E^{\prime }$ is equivalent elastic modulus; v isPoisson’s ratio.

Based on experimental data, the surface roughness and geometric shape of the bearing ring grooves underwent great changes after abrasive flow machining. Before machining, the higher surface roughness and irregularities led to a complex contact surface shape, resulting in a smaller curvature radius $R_{y}$ in the vertical direction in local areas, while the overall surface was relatively flat in the horizontal direction, leading to a larger $R_{x}$. This resulted in a smaller geometric parameter k, which in turn contributed to a reduced oil film thickness. After abrasive flow machining, the groove surface roughness was significantly reduced, and the texture became smoother, causing $R_{y}$ and the geometric parameter k to increase, ultimately resulting in an increase in the minimum oil film thickness. Furthermore, the material’s elastic modulus $E^{\prime }$ was increased with the introduction of residual compressive stress, leading to an increase in G, which further enhanced the minimum oil film thickness.

Rolling bearings operate under harsh conditions, often involving high speeds and high temperatures. Adding lubricants to the bearings can significantly reduce friction losses and carry away some of the generated heat; thus the operational lifespan of the shaft can be greatly enhanced. A sufficient thickness of the oil film can effectively separate the contact surfaces and withstand extremely high pressures to balance the external loads on the bearing. Therefore, the lubrication between the rolling elements and the raceway plays a crucial role in the bearing’s lifespan, fatigue wear, and friction [29].

## 4. Conclusions and Prospect

This study focuses on the abrasive flow polishing of aircraft engine main bearing rings. The surface roughness, residual stress, and oil film thickness of the bearing channels and oil holes were analyzed in detail and verified through experimentation. The research indicates that, through the reasonable design of the abrasive flow processing technique, the surface quality of the main bearings can be improved effectively and their performance in applications of aerospace engine can also be enhanced.

Firstly, by comparing the surface roughness of the bearing channels and oil holes before and after abrasive flow processing, results showed a significant reduction in roughness. This indicates that the abrasive flow polishing process can effectively enhance surface integrity and optimize the microtopography of the channels and oil holes, demonstrating excellent surface modification effects.

Secondly, the study examined the changes in residual stress of the main bearings before and after processing. The introduction of residual compressive stress during abrasive flow processing helps enhance the fatigue resistance of the material’s surface, contributing to the bearings’ durability under extreme conditions. These experimental results confirm the positive role of abrasive flow processing in improving the long-term operational stability and wear resistance of the main bearings.

Lastly, the analysis of oil film thickness revealed that, with the reduction of surface roughness, improvement of surface microtopography, and increase in residual compressive stress, the lubrication performance at the contact surface is significantly enhanced and the oil film thickness increases accordingly. These play crucial roles in improving the overall lubrication effect of the main bearings, reducing friction losses, and increasing lifespan of the bearings.

In summary, this research validates the effectiveness of abrasive flow polishing technology in enhancing the surface performance of aircraft engine main bearing channels and oil holes. The comprehensive impacts of the processing techniques on surface roughness, residual stress, and oil film thickness are clarified through experimental and theoretical analysis, which provide a scientific basis for subsequent process optimization.

## Figures and Tables

**Table 1:** Bearing ring raceway measurement record.

End Face Marking	Hardness	Measurement Points	Raceway Roundness/µm	Profile Accuracy/µm	Ra/µm	Rq/µm	Rv/µm	Rt/µm	Rz/µm	Surface Compressive Stress/MPa
1-1	HRC59.2	1	1.53	3.484	0.031	0.0397	0.127	0.5499	0.2479	312
		2		3.121	0.035	0.0462	0.1585	1.3189	0.2899	323
		3		3.442	0.030	0.0391	0.1293	0.6279	0.2447	301
		Mean Value		3.349	0.032	0.0417	0.1383	0.8322	0.2608	312
1-2	HRC61	1	1.79	1.944	0.033	0.0423	0.1594	0.9473	0.2737	316
		2		2.155	0.032	0.0423	0.1753	1.0019	0.2829	321
		3		1.838	0.032	0.0422	0.1697	1.007	0.2764	305
		Mean Value		1.979	0.032	0.0423	0.1681	0.9854	0.2777	314

**Table 2:** Abrasive characteristics composition table.

Materials	Mass Fraction [wt%]
Cubic boron nitride	30~70%
Styrene-butadiene rubber	20~50%
Binder	5~10%
Lubricant	3~8%
Dispersant	2~5%
Stabilizer	2~4%

**Table 3:** Abrasive flow machining experimental parameters table.

	Abrasive Medium	Machining Areas	Machining Time		Pressure in the Fixture Cavity/MPa		Machining Time
			Cycle Count	Single Cycle Time/s	Downward Cylinder Pressure	Upward Cylinder Pressure	Total Operating Time/s
1	5000-mesh cubic boron nitride	Oil holes	40	25	3.0~3.5	3.0~3.5	1000
2	10,000-mesh cubic boron nitride	Channels	60	30	3.0~3.5	3.0~3.5	1800
3	18,000-mesh cubic boron nitride	Channels	144	25	2.5~3.0	2.5~3.0	3600

**Table 4:** Roughness and inner diameter difference before and after oil hole machining.

Oil Hole Numbering	Surface Roughness of Oil Holes Before Machining Ra/µm	Surface Roughness of Oil Holes After Machining Ra/µm	Difference in Internal Diameter of Oil Holes Before and After Machining/mm
1	2.374	0.621	0.02
2	2.121	0.605	0.02
3	2.238	0.668	0.00
4	2.055	0.663	0.01
5	2.132	0.653	0.01
6	2.269	0.635	0.00
Mean value	2.198	0.641	0.01

**Table 5:** Measurement record of the main bearing raceway after testing.

End Face Marking	Value	Ra/µm	Rq/µm	Rv/µm	Rt/µm	Rz/µm	Surface Compressive Stress/MPa
1-1	Mean value	0.0199	0.0217	0.0692	0.4161	0.1304	−697.18
1-2		0.0192	0.0223	0.0841	0.4927	0.1388	−706.22

## Data Availability

All data generated or analyzed during this study are included in this published article.
